# Supplementary material for: Biomimetic peptide enriched nonwoven scaffolds promote calcium phosphate mineralisation
Source: RSC Adv. 2020 Jul 29;10(47):28332–42. doi: 10.1039/d0ra02446e (PMC9055731; doi:10.1039/d0ra02446e)
Supplement: RA-010-D0RA02446E-s001 [file RA-010-D0RA02446E-s001.pdf]

Electronic Supplementary Material (ESI) for RSC Advances.
This journal is © The Royal Society of Chemistry 2020

Supplementary information

SI Table 1. Reagents for preparation of SBF in the required order of dissolution.

Order	Reagent	Amount	Purity (%)	Formula weight (g/mol)
1	Sodium chloride ($NaCl$)	8.03	99.5	58.44
2	Sodium hydrogen carbonate ($NaHCO_3$)	0.35	99.5	84.00
3	Potassium chloride (KCl)	0.25	99.5	74.55
4	Di-potassium hydrogen phosphate trihydrate ($K_2HPO_4 . 3H_2O$)	0.23	99	228.22
5	Magnesium chloride hexahydrate ($MgCl_2 . 6H_2O$)	0.31	98	203.30
6	Hydrochloric acid solution (HCl) = 1 mol/L	39 (mL)	-	-
7	Calcium chloride ($CaCl_2$)	0.29	95	110.98
8	Sodium sulphate (Na_2SO_4)	0.07	99	142.04
9	Tris- hydroxymethyl aminomethane ($(HOCH_2)_3CNH_2$)	6.11	99	121.13

A

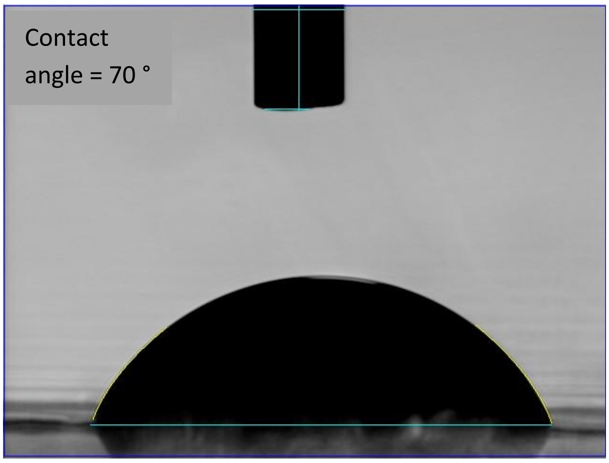

B

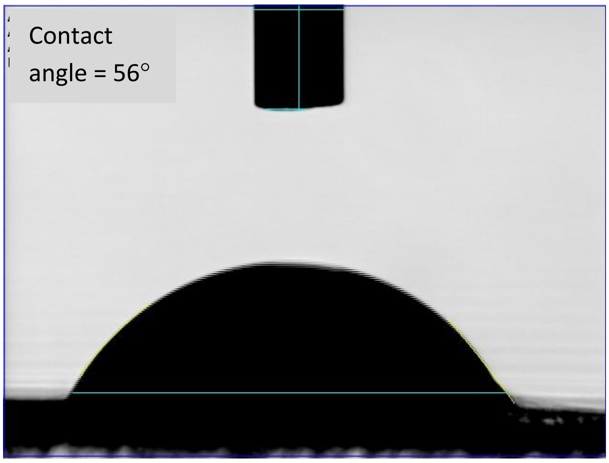

C

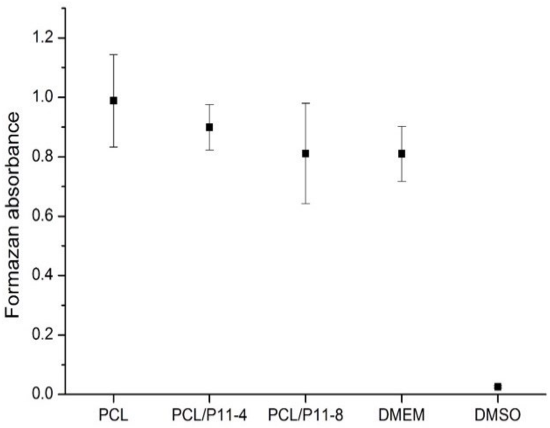

SI Figure 1. Initial contact angle of deionised water on electrospun scaffolds: (A-B) sample 2 and 3; PCL with 10 and 20 mg mL^{-1} P_{11}-4 respectively, showing increase in hydrophilicity with the addition of peptide. (C) OD absorption of fibrous samples 4 and 5 (PCL with 40 mg mL^{-1} of P_{11}-4 and P_{11}-8 respectively) and control samples at 570-650 nm correlated to the number of viable cells.

SI Table 2. Cell viability percentage of samples 4 and 5 (PCL with 40 mg mL^{-1} of P_{11}-4 and P_{11}-8 respectively) calculated based on OD absorption data. Blank is the the cell-free DMEM sample.

Sample	Mean OD (6 repetition)	Cell Viability % Based On Control 1	Cell Viability % Based On Control 2
**PCL/P11-4**	1.21	100.12	83.67
**PCL/P11-8**	1.12	92.08	76.95
**Blank**	0.05	-	-
**PCL (control 1)**	1.21	-	-
**Cell only (control 2)**	1.43	-	-
**Negative control**	0.10	-	-

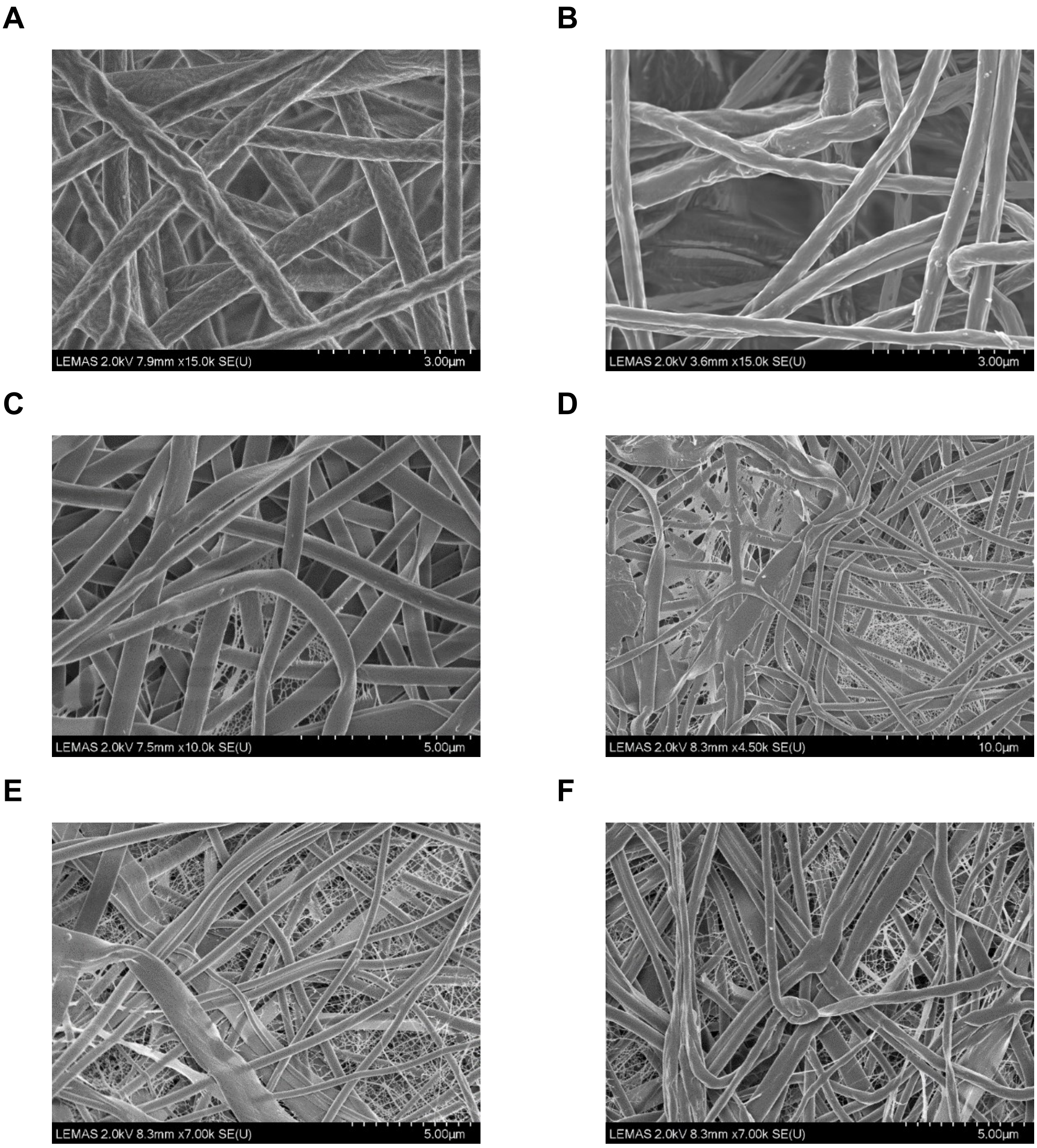

SI Figure 2. SEM micrographs of electrospun scaffolds after 168 hours incubation of (A-B) PCL at pH 3.5 and 10.5, (C-D) PCL/P_{11}-4 (40 mg mL^{-1}) at pH 3.5 and 10.5, and (E-F) PCL/P_{11}-8 (40 mg mL^{-1}) at pH 3.5 and 10.5.

SI Table 3. An example of elemental analysis of chemical elements in the P_{11}-4-supplemented scaffolds (with peptide concentration of 40 mg mL^{-1}) after immersion in SBF for 2 weeks obtained by SEM/EDX showing calcium and phosphorous atomic contents.

Element	Atomic Content (%)
C	37.07
O	40.54
Na	1.40
Mg	0.44
P	6.85
Cl	1.61
K	0.09
Ca	11.80
Cu	0.02
Ir	0.19
**Total:**	100.00

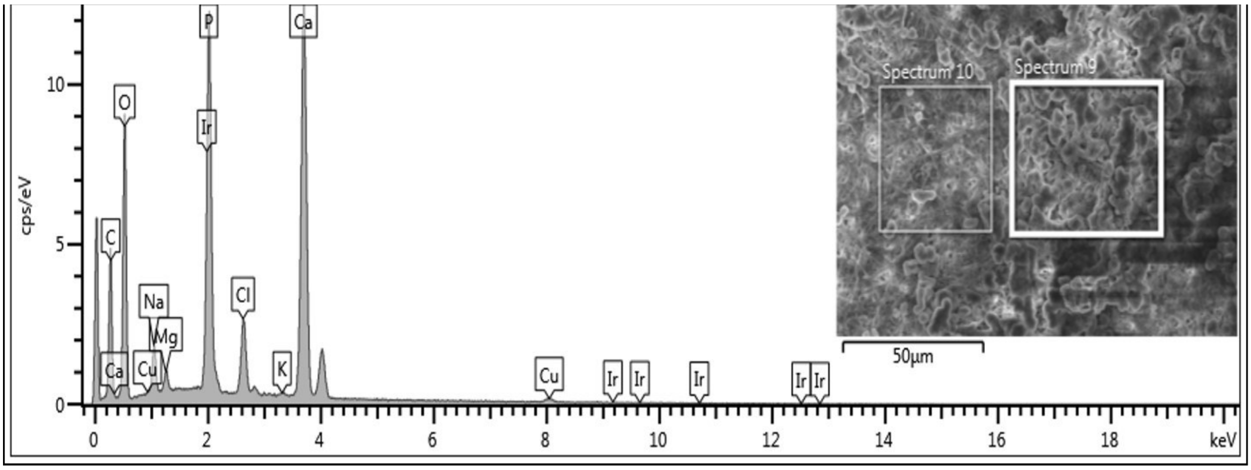

SI Figure 3. EDX spectra of P_{11}-4-supplemented scaffold (with peptide concentration of 40 mg mL^{-1}) immersed in SBF for 2 weeks showing calcium and phosphorous peaks.

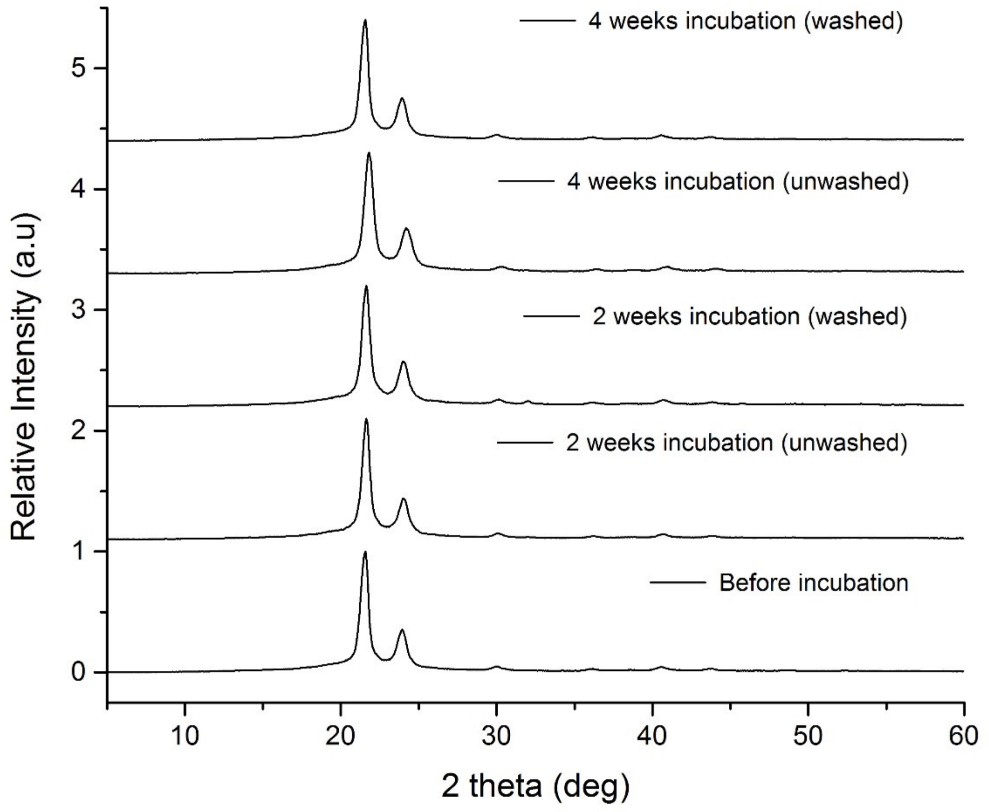

SI Figure 4. X-ray diffraction patterns of PCL control scaffolds before and after 2 and 4 weeks incubation in SBF (both washed and unwashed samples) showing no hydroxyapatite formation.
